# Adult Acquired Buried Penis: A Hidden Problem in Obese Men

**DOI:** 10.7759/cureus.13067

**Published:** 2021-02-01

**Authors:** Philip R Cohen

**Affiliations:** 1 Dermatology, San Diego Family Dermatology, National City, USA

**Keywords:** acquired, adult, buried, genital, hidden, men, rejuvenation, obesity, penis, scrotal

## Abstract

Buried penis is a condition where the penis is hidden by abdominal or suprapubic skin or fat. In adult men, buried penis occurs as an acquired condition most commonly caused by morbid obesity. The clinical characteristics of three obese men with adult acquired buried penis are described and the associated features of the buried penis are reviewed. In addition to morbid obesity, a buried penis can result from other etiologies, such as hidradenitis suppurativa, iatrogenic causes such as elective surgeries, infections, lichen sclerosus, penoscrotal lymphedema, and traumatic events. Lower urinary tract symptoms, such as voiding, and post-voiding problems are the most common presenting complaints; however, bacterial and fungal infections, phimosis, psychological issues, and sexual dysfunction, are also buried penis-related symptoms. The evaluation of a man with adult acquired buried penis begins with a detailed history for condition-related symptoms. Examination of the patient, both standing and supine with an attempt to demonstrate the penis using digital compression of the surrounding skin and fat, should be performed to determine the extent of the problem and whether comorbid conditions-such as infection and lichen sclerosus-are present. Both buried penis and lichen sclerosus can predispose to the development of penile squamous cell carcinoma; the diagnosis of this tumor can be delayed in men with adult acquired buried penis since an adequate penile examination is difficult or impossible. A multidisciplinary approach-including surgeons, primary care physician, registered dietitian nutritionist, and psychiatrist-should be considered for a patient with a buried penis. The surgical management is individualized and based on not only the extent of the problem but also whether an associated condition, such as urethral stricture, is present. Most patients are pleased with the functional and aesthetic outcome following surgery.

## Introduction

Obesity is a prevalent health problem of epidemic proportion. Indeed, a large portion of the population is overweight or obese. There are several obesity-associated medical conditions, such as diabetes mellitus and hypertension; in addition, adult acquired buried penis is a comorbid problem that may develop in obese men [1-4].

Buried penis describes a condition in which either skin and/or fat in the prepubic area encompasses a penis of normal size; clinically, the penis is not visible or appears short and buried in the prepubic tissue when the patient is examined. Buried penis can be a congenital condition or an acquired disorder; it occurs in children and adults who have either a normal or an elevated body mass index. There are multiple potential causes of acquired buried penis; however, obesity is the most common etiology in adult men [1-16].

The features of buried penis in three obese adults are described. One of the men also had biopsy-confirmed penile lichen sclerosus. The characteristics of adult acquired buried penis-a hidden problem in obese men-are reviewed.

## Case presentation

Case 1

An obese 62-year-old man presented for a total body skin examination. His past medical history was significant for diabetes mellitus, heart disease, hyperlipidemia and hypertension. His cutaneous history had been remarkable for benign pigmented purpuric eruption of his legs, intertrigo of his groin region, onychomycosis, actinic keratoses, and squamous cell carcinoma on his right forearm.

**Figure 1:**
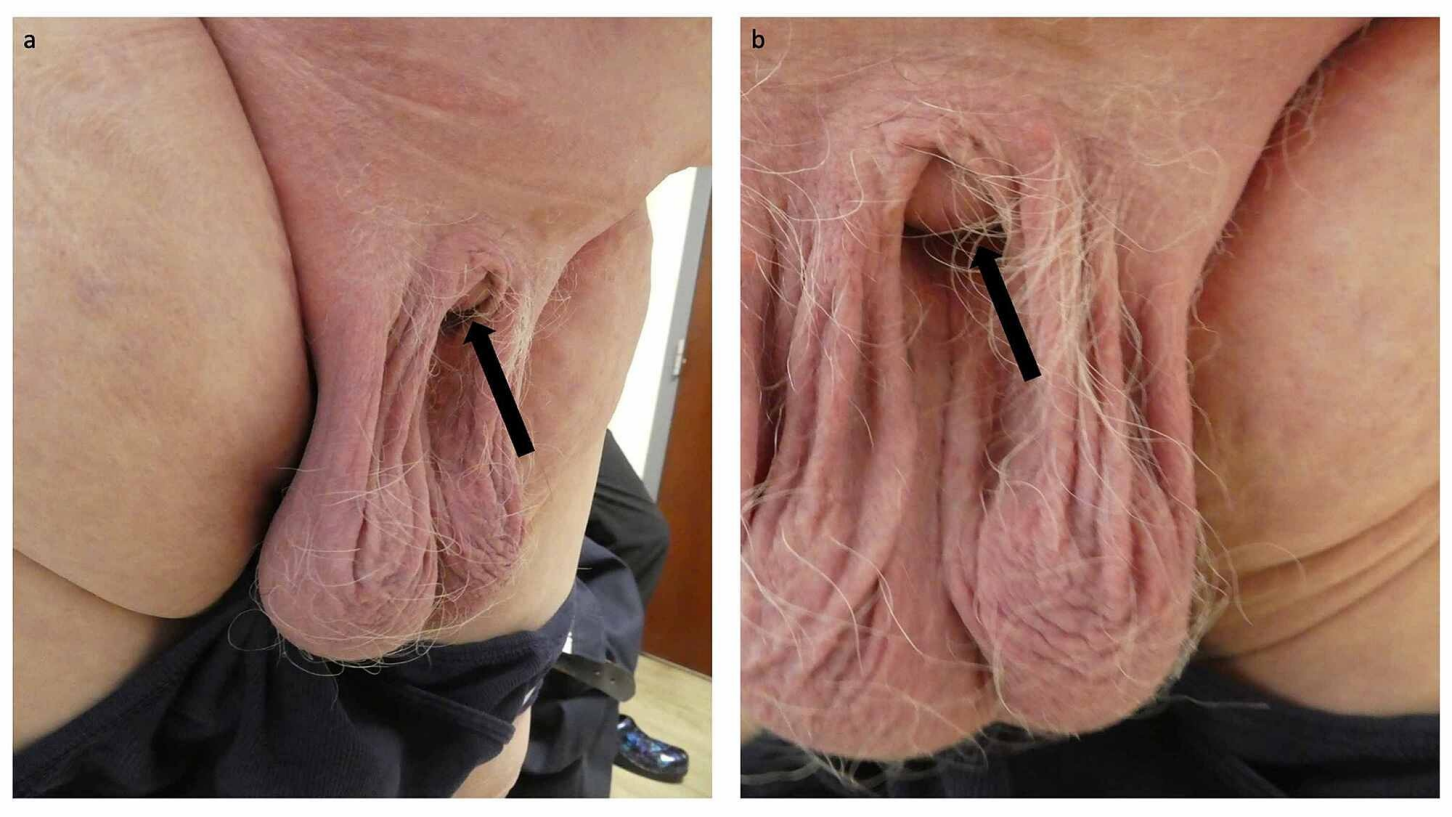
Adult acquired buried penis in a 62-year-old obese man with condition-associated recurrent Candida intertrigo Distant left (a) and closer frontal (b) views of the genital region of an obese 62-year-old man. Only the distal tip of his circumcised buried penis was visible (black arrow). The Candida intertrigo affected not only the skin fold beneath the lower abdomen but also the inguinal region and groin.

Cutaneous examination showed new actinic keratoses presenting as keratotic plaques on his arms, face, and scalp. He had recurrence of his intertrigo; the candidiasis appeared as confluent erythematous patches with minimal scaling on his bilateral inguinal grooves, the skin fold beneath his lower abdomen, and his groin. His pubic region demonstrated scrotal alopecia and scrotal laxity; his circumcised penis was buried and only the tip of the glans was visible (Figure 1).

His actinic keratoses were treated with cryotherapy using liquid nitrogen. His recurrent fungal intertrigo, a buried penis-related comorbidity, was successfully managed by applying ketoconazole 2% cream twice daily. He did not want to pursue any treatment for his adult acquired buried penis.

Case 2

An obese 71-year-old man presented for a total body skin examination. His past medical history was significant for diabetes mellitus, gastroesophageal reflux disease, hyperlipidemia, hypertension, and low grade papillary urothelial bladder carcinoma. His cutaneous history had been remarkable for stasis dermatitis of his legs, onychogryphosis and pincer nails of the toenails on both feet, and a basal cell carcinoma on his right proximal nasal bridge near the medial canthus.

**Figure 2:**
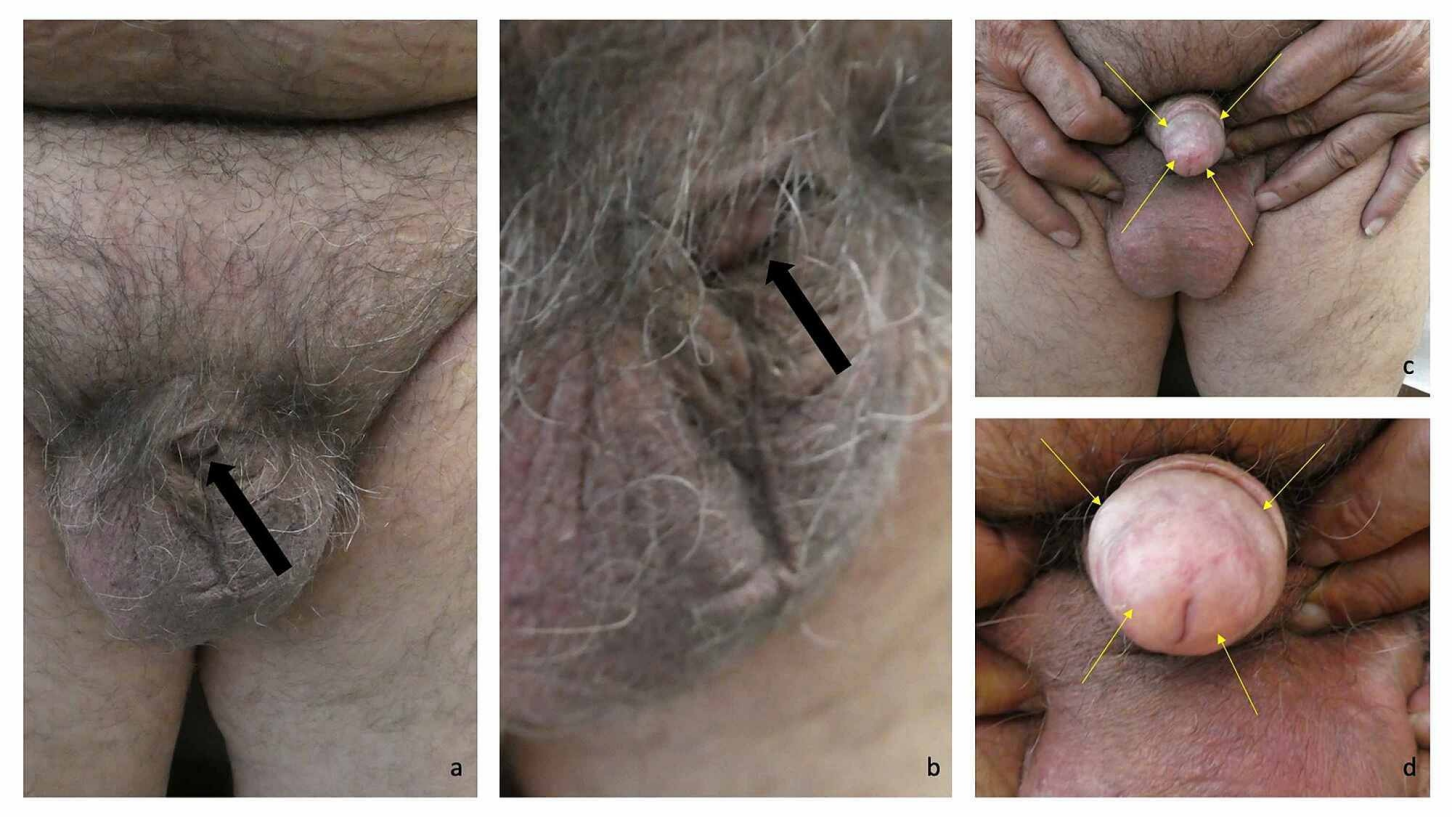
Adult acquired buried penis in a 71-year-old obese man with condition-related lichen sclerosus Distant frontal (a) and closer left (b) views of the genital region of an obese 71-year-old man only show the distal tip of his circumcised buried penis (black arrow). After pressure was applied with fingers to the pubic area above his scrotum, his distal penile shaft and glans penis were able to be demonstrated (c and d). His glans penis showed lichen sclerosus (c and d); it was white, indurated, and sclerotic (yellow arrows). Treatment was initiated with halobetasol 0.05% ointment.

Cutaneous examination of his pubic region demonstrated not only scrotal alopecia, but also that his circumcised penis was buried and only the tip of the glans penis was visible (Figure 2). His original circumcision was five years ago and he had recently had another circumcision two months ago; the submitted tissue from the recent procedure was diagnosed as lichen sclerosus. He was able to show his distal penile shaft and penis by applying pressure to the surrounding pubic areas with his fingers. His indurated sclerotic glans penis had several white areas.

Correlation of his history and examination established diagnoses of obesity-associated adult acquired buried penis and lichen sclerosus. He had been applying ketoconazole 2% cream to his glans penis without improvement. Halobetasol 0.05% ointment twice daily was prescribed to be applied to his penile shaft and glans penis. Treatment for his adult acquired buried penis was not desired.

Case 3

An obese 87-year-old man presented for a total body skin examination. His past medical history was significant for arthritis, diabetes mellitus, hypertension, and seasonal allergies. His cutaneous history had been remarkable for actinic keratoses, dermatitis on his lower back and leg, basal cell carcinomas on his nasal bridge and chest, and a squamous cell carcinoma on his right cheek.

**Figure 3:**
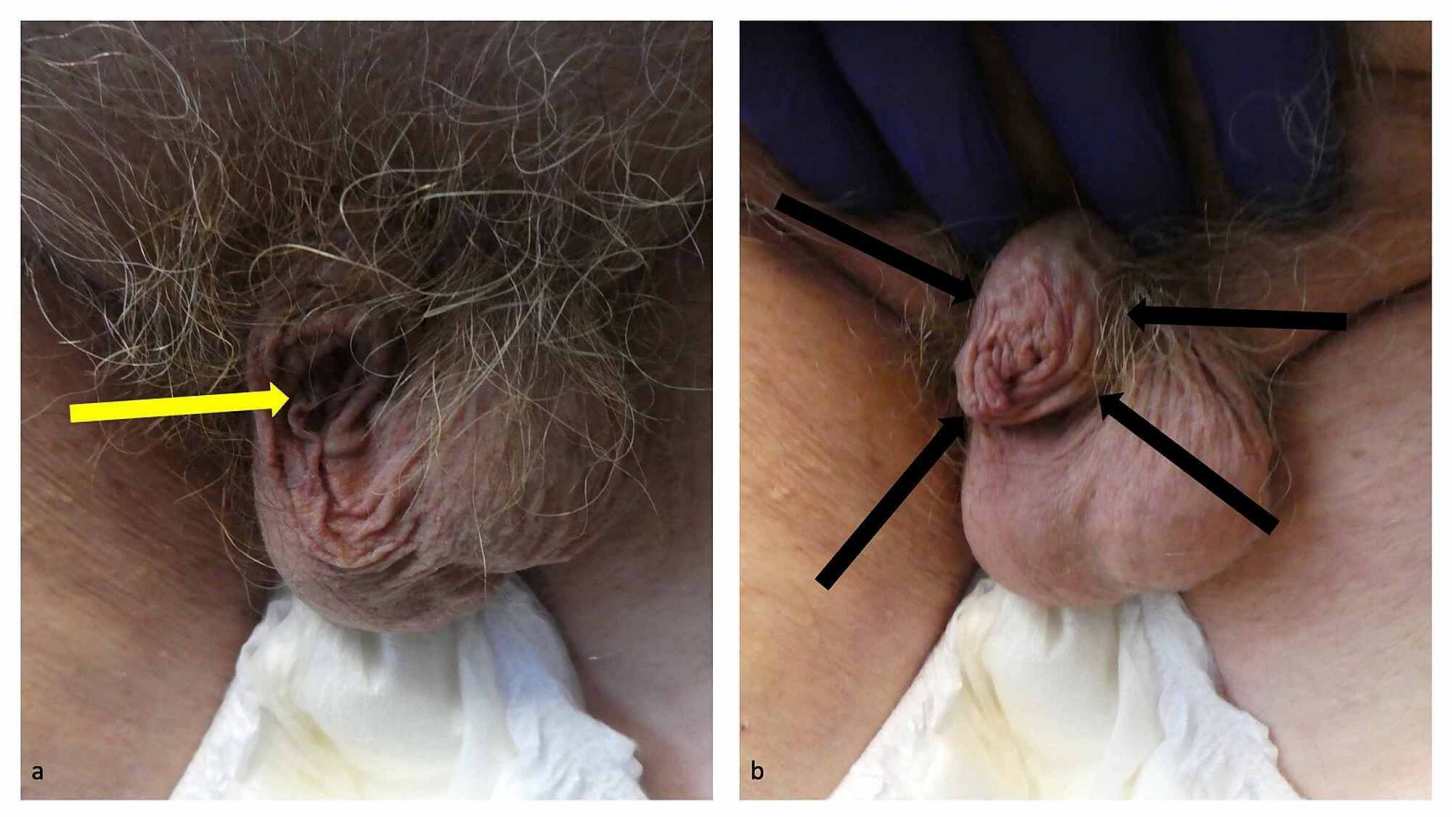
Adult acquired buried penis in an 87-year-old obese man An obese 87-year-old man’s adult acquired buried penis cannot be observed; however, a yellow arrow shows where his hidden uncircumcised penis was located (a). The distal portion of his penis was able to be demonstrated after pressure had been applied to the suprapubic fat (escutcheon); the black arrows demarcate his proximal penis (b).

Cutaneous examination showed keratotic plaques on his arms and scalp and xerosis on his lower back and legs; the actinic keratoses were treated with cryotherapy using liquid nitrogen. He has scrotal alopecia and his uncircumcised penis was completely hidden (Figure 3). After pressure was applied to his suprapubic area, the distal portion of his penis was able to be observed. He did not want any treatment for his adult acquired buried penis.

## Discussion

The term buried penis was introduced by Edward Loughborough Keyes, MD in 1919 [2,5,15]. It is a progressive and debilitating problem. Subsequently, the condition has been referred to as concealed, hidden, inconspicuous, trapped, and vanishing penis [1-2,5,6,8,16].

Several etiologies have been associated with the development of buried penis. In children, buried penis has primarily resulted from two causes. These include poor penile skin suspension secondary to abnormal mobility of the dartos fascia, or inadequate attachment to Buck facia, or both [2,10,11,15].

In adults, there are several possible causes of acquired buried penis. In addition to morbid obesity, some of the causes of buried penis are hidradenitis suppurativa, iatrogenic etiologies (such as the following circumcision or after aesthetic surgery including, but not limited to, penile and scrotal enlargement), infections (such as those resulting from bacterial and fungal organisms), lichen sclerosus, penoscrotal lymphedema (secondary to multiple etiologies including arteriobifemoral bypass grafting, inguinal lymphadenectomy for either penile cancer or melanoma, lymphatic filariasis--commonly known as elephantiasis--in men living in developing countries, and prostatectomy with accompanying pelvic lymph node dissection), and traumatic events. Wearing tight underwear can also potentially result in bacterial or fungal infection of the glans penis; the balanitis can promote the development of buried penis [1,3,5,6].

Buried penis in adults is a common problem that is increasingly being seen by reconstructive urologists. Indeed, 87% of the men who underwent surgical treatment for buried penis were obese. However, based on the increased prevalence of obesity, there seems to be a paucity of the literature on buried penis [1,5,6].

Originally, a classification of buried penis in children was established [10,11]. However, it was not adapted to men with adult-onset buried penis. Eventually, several groups of investigators have proposed classifications for adult acquired buried penis based on the patient’s clinical presentation and the subsequent management of the man depended on the classification subtype of his buried penis [2,3,7-9,16].

Tausch et al. introduced a classification system for adult acquired buried penis in 2016. Grade 1 patients had viable penis skin, whereas grade 2 patients had nonviable penile skin and grade 3 patients had genital lymphedema. Treatment included phalloplasty with or without a scrotal flap for grade 1 patients, replacement of the denuded skin with a split-thickness skin graft for grade 2 patients, and excision of involved tissues and any appropriate adjunctive procedures followed by split-thickness skin grafting [3].

Subsequently, in 2018, three additional classification systems were proposed. Mirastschijski's novel classification system of adult acquired buried penis included type 1 (pseudo-buried penis in which excessive prepubic soft tissue covers the penis without invagination), type 2 (intermediate type buried penis in which there is partial invagination of the penile shaft with excess prepubic soft tissue but manual extrusion of the penis is possible), and type 3 (classic buried penis in which there is complete invagination of the penile shaft with a massive prepubic fat cushion and no manual extrusion of the penis is possible). Type 1 required apronectomy and a prepubic lift; types 2 and 3 both required apronectomy and penile anchoring sutures-however, type 3 also needed skin grafts or local flaps for penile shaft reconstruction [2].

In contrast, Pariser et al. proposed a classification system based on the complexity of the surgical repair. There are five categories; patients requiring more than one procedure were assigned to the highest category. The surgical complexity ranged from penile unburying with a local skin flap (category I) to abdominal panniculectomy (category V). In between, the procedures included penile skin grafting (category II), scrotectomy or scrotoplasty (category III), or excutcheonectomy (category IV) [9].

Finally, the Wisconsin classification (described by Hesse et al.) stratified patients with adult acquired buried penis according to preoperative examination findings. Type 1 men had a buried penis secondary to skin deficiency, iatrogenic scarring, and/or diseased penile skin; treatment included peri-penile skin excision and split-thickness skin grafting. Type II men with buried penis had excessive abdominal and/or suprapubic fat with lymphedema but no penile skin deterioration; they were treated with panniculectomy, or escutcheonectomy, and/or suprapubic debulking followed by suprapubic suspension. Type III buried penis patients also had excessive abdominal and/or suprapubic fat with lymphedema and penile skin deterioration; in addition to penile exhumation and peri-penile skin excision, they were treated with panniculectomy, or escutcheonectomy, and/or suprapubic debulking followed by split-thickness skin grafting. And type IV patients had excessive abdominal and/or suprapubic fat with penile skin deterioration and severe scrotal edema; their treatment not only included panniculectomy and/or escutcheonectomy, but also penis exhumation and peri-penile skin excision with split-thickness skin grafting in addition to scrotectomy and translocation of the testes [7].

The most common presenting complaints of patients with adult acquired buried penis are lower urinary tract symptoms. These predominantly include both voiding difficulties such as inability to control urination (secondary to hesitancy and poor flow) and post-voiding problems such as dribbling and incontinence. In addition, other symptoms include bacterial and fungal infection of the soft tissues and urinary tract, phimosis, psychological issues (such as depression and low self-esteem) and sexual function issues (such as erectile dysfunction and painful or impossible sexual activity secondary to the buried phallus) [1,7].

The evaluation of a man with adult acquired buried penis should include a detailed history focused on the duration of time that the penis has been buried and whether the occurrence of the vanishing penis was associated with either gaining or losing weight. Inquiry should be made regarding not only symptoms associated with voiding problems or sexual dysfunction, but also the presence of absence of benign prostatic hypertrophy, injections for genital enlargement, prior surgeries (such as circumcision, lymph node dissection, and penis enlargement), and urethral stricture. In addition, evaluation for potentially associated conditions, such as diabetes mellitus, hidradenitis suppurativa, hypertension, obesity, pulmonary issues (related to obesity), and urologic problems, should be conducted [3,4,6,8].

A man who is being evaluated for adult acquired buried penis should be examined both standing and supine; either the abdominal panniculus or the suprapubic fat pad (escutcheon) or both may be covering the penis. The penis should be examined to determine whether it has been circumcised and the penile meatus should be inspected for stenosis; in addition, the penis and scrotum and suprapubic region should be assessed for lymphedema, thickening of the skin, and whitening of the skin associated with lichen sclerosus [6,8].

The skin and fat surrounding the penis should be compressed by the evaluator’s fingers to determine if the penis can be demonstrated. Overabundance of escutcheon or phimosis may prevent deliverance of the penis. Also, the presence of scrotal enlargement may be a clue to associated hidradenitis suppurativa [2,6,7].

Voiding symptoms in men with buried penis may be secondary only to the hidden penis. However, lichen sclerosus or urethral stricture or both may result in voiding symptoms. Although urethral strictures are frequently observed in men with adult acquired buried penis, an etiologic association between these conditions has not been established; however, the presence of lichen sclerosus in adult acquired buried penis patients who also have distal penis (fossa navicularis and/or meatus) urethral strictures may share a common pathogenesis related to the lichen sclerosus that predisposes to both buried penis and urethral strictures. Cystoscopy, with or without imaging studies (such as a retrograde urethrogram), may be necessary to establish the diagnosis of urethral stricture [3,6,8,9].

There are several comorbid conditions associated with buried penis; the disorders can have an etiologic role in the development of a hidden penis and/or be a sequelae of the buried penis. Poor hygiene and trapped moisture results in bacterial and fungal colonization and infection with subsequent maceration of the penile shaft tissue. Lichen sclerosus can also promote or result from a buried penis [6,8,9,16-20].

Lichen sclerosus (also referred to as lichen sclerosus et atrophicus and balanitis xerotica obliterans) presents as white fibrotic induration of the glans penis, penile shaft and/or foreskin (if present) with possible phimosis; it is characterized microscopically by a band-like lichenoid lymphocytic infiltrate in the upper dermis, often with vacuolar alteration of the basal layer of the atrophic epidermis with overlying hyperkeratosis. Medical management predominantly involves the topical application of ultra-high potency corticosteroids; however, recently a potential role for intralesional platelet-rich plasma has been proposed. Exacerbation or recurrence of buried penis can be a postoperative complication of circumcision of the lichen sclerosus-affected tissue [17-20].

One of the reported patients with adult acquired buried penis had lichen sclerosus. The diagnosis of lichen sclerosus was delayed as a result of the difficulty his clinicians had in examining his penis. Although he had previously been circumcised, when he eventually presented to a urologist the affected tissue was surgically removed; subsequently, the residual disease on his glans penis was managed medically with twice daily application of halobetasol 0.05% ointment.

Squamous cell carcinoma can develop in the setting of either buried penis or lichen sclerosus [12-14]; a systematic review estimated that men with lichen sclerosus had a 2%-8% life-time risk of their lichen sclerosus transforming to squamous cell carcinoma [17]. In a retrospective study of 150 patients with adult acquired buried penis, 11 men (7%) had penile squamous cell carcinoma; despite the decreased incidence of invasive penile cancer in men who have undergone neonatal circumcision, four of the men with a buried penis in whom cancer was discovered had been circumcised as a neonate or child [12]. A similar observation was also made by other investigators [14]. Similar to one of the men described in this paper, since an adequate examination of the penis is difficult or impossible to perform in men with adult acquired buried penis, the diagnosis of lichen sclerosus or squamous cell carcinoma or both may be delayed [12-14].

The management of adult acquired buried penis is based upon the etiology and the extent of disease involvement as determined by the clinical presentation of the patient. Ideally, there should be a multidisciplinary approach to the patient. This would include not only the surgeons (such as the urologist, bariatric, and plastic-reconstructive specialists) but also the primary care physician, registered dietitian nutritionist, and psychiatrist [2].

The specific surgical intervention must be determined for each individual patient. However, general concepts for the management of adult acquired buried penis include mobilization of the penis (by the release of tethering bands and scars), fat removal (of the abdominal panniculus, the suprapubic fat pad, or both), appropriately suspending the base of the penis (to prevent retraction), and restoring penile skin if the penis has been denuded; when replacement penile tissue is necessary, recommendations vary with regards to using split-thickness skin grafts, thick split-thickness skin grafts and/or full-thickness skin grafts. Scrotectomy may also be necessary for some of the men with adult acquired buried penis [1-9,15,16].

Postoperative complications may occur following surgical treatment of buried penis. Some of these include abnormal scarring, granuloma formation, paradoxical penile shortening, penile deformity, psychological sequelae, and sexual dysfunction. However, most of the men are pleased with the functional and aesthetic outcome following surgical management of their adult acquired buried penis [5,9].

## Conclusions

Obesity is the major cause for adult acquired buried penis. A buried penis is a normal sized penis that either appears short or is hidden from view by abdominal or suprapubic skin or fat. Three older men who had adult acquired buried penis are described; their buried penis was an incidental finding during a total body skin examination. Prior to their cutaneous evaluation, the men did not realize that there were surgical interventions that might be useful to remedy their buried penis; however, perhaps because of their age or the chronicity of their problem or both, none of the patients desired to have their buried penis further evaluated or treated. There are several condition-related symptoms (such as bacterial and fungal infections, phimosis, psychological issues, sexual dysfunction, and voiding and post-voiding problems) and disorders (such as lichen sclerosus) that can be present in men who have a hidden penis; urethral strictures are also commonly present in patients with adult acquired buried penis. In addition, both buried penis and lichen sclerosus can be associated with the development of penile squamous cell carcinoma. The optimal management of adult acquired buried penis is multidisciplinary; surgical intervention is often successful in providing a functional and aesthetic outcome.
